# Quality of Life and Sexual Satisfaction in Women with Breast Cancer Undergoing a Surgical Treatment and in Their Male Partners

**DOI:** 10.3390/jcm11236960

**Published:** 2022-11-25

**Authors:** Renato Martins, Patricia Otero, Ángela J. Torres, Fernando L. Vázquez

**Affiliations:** 1 EMC Euromentalcare (Especialistas en Saúde Mental), 4780-568 Santo Tirso, Portugal; 2 Department of Psychology, University of A Coruña, 15071 A Coruña, Spain; 3 Department of Psychiatry, Radiology, Public Health, Nursing and Medicine, University of Santiago de Compostela, 15782 Santiago de Compostela, Spain; 4 Department of Clinical Psychology and Psychobiology, University of Santiago de Compostela, 15782 Santiago de Compostela, Spain

**Keywords:** breast cancer, surgical treatment, quality of life, sexual satisfaction, partner

## Abstract

This study aimed to determine the quality of life and sexual satisfaction in a sample of 389 women with breast cancer who underwent a surgical treatment and 366 men who were these women’s partners. The sample was recruited from the Portuguese League Against Cancer by 10 trained psychologists who assessed the quality of life and sexual satisfaction of the participants. Data on the sociodemographic variables, diagnosis and treatment in the female participants, relationship with their partner, anxiety and depression, and body image were also collected. It was found that 76.6% and 54.2% of the women had low physical and mental health, respectively, while 100% of partners had acceptable physical and mental health. The predictors of women’s physical health were months since surgery, current treatment, completed treatments, satisfaction with the current relationship with their partner, lower anxiety and depression, and better body image. The predictors of women’s mental health were months since diagnosis and treatment completion, satisfaction with partner support during the illness, lower anxiety and depression, and better body image. The predictors of both physical and mental health of partners were lower anxiety and depression. In addition, 88.4% of women and 100% of partners presented with sexual dysfunction. The predictors of women’s sexual satisfaction were being older, satisfaction with their relationship with their partner before the illness, lower anxiety and depression, and better body image. The predictors of sexual satisfaction of the male partners were psychological/psychiatric support, satisfaction with their current relationship with their partner, and lower anxiety and depression. These findings suggest that interventions targeted at the quality of life of women and sexual satisfaction with a couple perspective are needed.

## 1. Introduction

Currently, cancer is one of the most prevalent diseases in the world, with 32 million people diagnosed and about 8.2 deaths annually, according to the International Agency for Research on Cancer [1]. More specifically, breast cancer is the most common type of cancer in women, encompassing over 25% of all diagnosed oncological pathologies [2]. Within the last five years, 6,875,099 women were diagnosed worldwide, with 1,814,572 of those diagnoses being in Europe [3]. In Portugal, breast cancer has the highest incidence rate of all cancers, accounting for one third of the tumors diagnosed in women [4]. It is the second leading cause of death in the country, after cardiovascular disease [5], and the main cause of early deaths (i.e., deaths before the age of 70) [4].

Despite these concerning statistics, advances in medicine, including early diagnoses and more effective treatments, have increased the survival rates of breast cancer. The survival rate in Portugal is 85% at 5 years post-diagnosis [4]. Among the possible treatments, surgery (or mastectomy, the removal of the entire breast), combined with radiotherapy or chemotherapy, continue to be the therapeutic modalities that offer a higher probability of cure [6]. Nevertheless, it presents inevitable postoperative side effects (e.g., pain, possible bleeding, bruising, infection, and lymphedema) and self-image changes [7]. As a result, there is a tendency to perform surgeries that involve greater preservation of breast tissue, followed by breast reconstruction techniques.

Due to the diagnosis and the associated treatments of breast cancer, profound psychological and physical consequences often result. These consequences often have an impact on the quality of life of women [8,9], and their sexual functioning and satisfaction [10,11]. Previous research has found that the variables related to quality of life of women with breast cancer were age [12,13]; current treatments with associated side effects, such as chemotherapy or radiotherapy [14]; level of family support [15]; symptoms of anxiety and depression [16]; and body image [7]. The variables associated with sexual satisfaction were age [17,18,19], chemotherapy and hormonal treatments [20], relationship satisfaction with their partner [10], symptoms of anxiety and depression [21], and body image [22,23].

While the priority for research has been targeted towards women who are recovering from breast cancer, there is a recognition that their partners may also be considerably impacted [24]. Male partners can experience anxiety, fear, and difficulty adapting to the evolving body image of their female partners; these factors can affect their own quality of life and sexual satisfaction. Previous research has found that depression and anxiety are closely related to the quality of life and sexual satisfaction of the partners of women with breast cancer [25,26].

The majority of previous research has assessed the consequences of breast cancer diagnosis and treatment on the quality of life and sexual satisfaction in women, but there is a dearth of research on women undergoing surgical treatment specifically. Additionally, research on the quality of life and sexual satisfaction of male partners and their associated variables are even more rare [10,25,26].

Thus, the aim of the current study was to determine the quality of life and sexual satisfaction of women with breast cancer who have undergone surgical treatments and to identify the associated sociodemographic and clinical variables. Furthermore, an additional aim was to examine the quality of life and sexual satisfaction of the male partners and to identify the associated sociodemographic and clinical variables.

## 2. Materials and Methods

### 2.1. Participants

A cross-sectional study was conducted. Women treated surgically for breast cancer, who were or are being followed by the Portuguese League Against Cancer (PLAC) and their male partners were recruited. The inclusion criteria of women were (a) being 18 years of age or older, (b) having undergone surgical treatment due to breast cancer, (c) having a male partner at the time of the study, and (d) sharing daily life or having regular contact with their partner. The inclusion criteria of the male partners were (a) being 18 years of age or older, (b) having a female partner at the time of the study who had undergone surgical treatment due to breast cancer and who meet the eligibility criteria to participate in the present study, and (c) sharing daily life or having regular contact with their partner. Women and their partners were excluded from the current study if they (a) could not read and/or write, (b) changed their address during the study period, (c) had a mental disability or severe neurological impairment that seriously impaired their interaction/understanding, (d) had uncontrolled pain symptomatology, (e) had a terminal illness, and (f) had not given their informed consent.

To standardize the data-collection process, an evaluation protocol was developed, 10 clinical and health psychologists were trained through 8 h of theoretical and practical seminars, and a pilot study was conducted to evaluate the degree of applicability of the protocol. Subsequently, a meeting was held with the PLAC service officials to organize contact with the sample. The evaluation instruments were administered individually and in groups at the end of the PLAC consultation by the team of 10 psychologists trained for this purpose.

A total of 996 people were invited to participate in the current study: 536 women and their heterosexual partners if they had partners, which was the case for 460 women. It was found that 127 of the women and 74 of the male partners did not meet the eligibility criteria. As a result, 795 participants (409 women and 386 male partners) met the eligibility criteria and were invited to complete the assessment instruments. The response rate was 95.0%; 40 people (20 women and 20 men) declined to participate, making up 5.0% of the total sample. Thus, a final sample consisted of 755 participants: 389 women with breast cancer underwent surgical treatment and 366 male partners of these women.

All participants gave their informed consent. Participation was voluntary, without monetary or other incentives. The study was conducted in accordance with the latest revision of the Helsinki Declaration; was approved by the Ethics Committee of the Faculty of Medicine of the University of Porto (protocol code: PCEDCSS-FMUP 06/2016); and was authorized by the delegations, units, and/or extensions of the Service of Psychology and Oncology (protocol code: PO-LPCC-NRN-RSEV-03/2016), the Winning and Living Movement (protocol code: MVV-RD-01/2016), and the Project Evaluation Commission of the PLAC (protocol code: PO-LPCC-NRN-RSERV-03/2016).

### 2.2. Instruments

For the evaluation of sociodemographic characteristics, diagnosis and treatment, and relationship with their partners, an ad hoc sociodemographic questionnaire was used for this study. To evaluate anxiety and depression, the Hospital Anxiety and Depression Scale (HADS; [27]; Portuguese version of Pais-Ribeiro et al. [28]) was used. It consists of 14 items with 4-point Likert response options, which are divided into two subscales: Anxiety and Depression, with a score of 0 to 21 on each subscale. Higher values indicate higher levels of anxiety and depression, with a cut-off point of 8 on each subscale. It has a Cronbach’s alpha of 0.76 on the anxiety subscale and of 0.81 on the depression subscale. To evaluate body image, we used the Body Image Scale (EIC) built on the basis of the Body Image Scale of Hopwood [29] and validated by Palhinhas et al. [30]. It consists of 14 items where the answers are obtained on a 4-point Likert scale and the final score can vary between 14 and 56, and it is interpreted here that lower values mean worse relation with body image and higher values better relation with body image. It has a Cronbach’s alpha of 0.90.

To assess health-related quality of life, the Medical Outcomes Study, Short Form (SF-36; [31]; Portuguese version of Ferreira [32]) was used. It consists of 36 items, which are distributed in eight dimensions (Physical Function, Physical Performance, Body Pain, General Health, Vitality, Social Function, Emotional Performance, and Mental Health), one item of health evolution, and two summary measures (Physical Health and Mental Health). Each scale can have a score from 0 to 100, and high results correspond to a good state of health and vice versa. The Portuguese version of the SF-36 has a Cronbach’s alpha value of 0.70 or more in all dimensions. To assess sexual satisfaction, the Golombok–Rust Inventory of Sexual Satisfaction (GRISS; [33]; Portuguese version by Vilarinho and Nobre, [34]). This instrument consists of two versions, one female and one male, both with 28 items, answered on a 5-position Likert scale. The total score has amplitude between 28 and 140 points, and is transformed using a 9-point scale, in which higher scores reveal more problems and scores of 5 or more are considered indicators of sexual dysfunction. The female version features a Cronbach’s alpha of 0.86, and the male version features a Cronbach’s alpha of 0.94.

### 2.3. Data Analysis

To evaluate the socio-demographic and clinical variables associated with diagnosis and treatment, regarding involvement, psychological comorbidity, and body image, we used the mean (*M*) and standard deviation (*SD*) in the case of quantitative variables, and absolute (*n*) and relative (%) frequencies in qualitative variables. There were made bivariate analysis using Student’s *t*-test for independent samples, ANOVA, followed by post hoc Scheffé test, Pearson’s r correlation, and Spearman’s rho correlation. Subsequently, linear multivariate regressions analyses were performed to evaluate the association between quality of life (physical and mental health, women and men) and sexual satisfaction (women and men) with variables that were previously significant in the bivariate analyses. Statistical analyses were performed using the SPSS version 20.0. All significance tests were two-tailed, and a 5% significance level was assumed.

## 3. Results

### 3.1. Characteristics of the Participants

As can be seen in Table 1, women with breast cancer who underwent surgical treatments had an average age of 50.6 years (*SD* = 12.6), and the majority (54%) had between a first and second cycle of studies, had employment (61.7%), were married (97.4%), had children (83.0%), and had an average of one child (*SD* = 0.9). The partners had an average age of 53.7 years (*SD* = 12.2), and the majority (61.7%) had a first or second cycle of primary education and were employed (58.7%).

We found that the women had been diagnosed, on average, 53.6 months ago (*SD* = 24.2) and underwent surgery, on average, 47.4 months ago (*SD* = 22.0). The majority (68.1%) underwent mastectomy and breast reconstruction, whose mean time of breast reconstruction was 18 months (*SD* = 24.7). For the initial cancer treatments, the majority (62.0%) had a combination of several treatments. The majority (72.0%) reported having already completed the treatments, on average, 28.2 months ago (*SD* = 23.7). For those who have not yet completed it, the current treatment was mostly (12.6%) chemotherapy. When analyzing the main side effects resulting from the treatments, the most common (71.2%) was hair loss. It was found that 58.6% were currently in menopause and 73.0% had sought out psychological/psychiatric support. Regarding the clinical data of the partners, it was verified that 18.6% had sought out psychological/psychiatric support.

The relationship and therapeutic involvement variables showed that 73.8% of women considered that before the disease, they had a moderately or very satisfactory relationship with their male partner; 63.8% reported a moderately or very satisfactory current relationship; 57.6% of women reported that the support received by their partner during the disease was moderately or very satisfactory. The majority (84.3%) did not address sexuality with health professionals; 74.3% were not involved in the choice of treatments, and 80.7% were not able to share their experience with other women. In the partners, it was found that 53.5% considered that before the disease, they had a moderately or very satisfactory relationship with the woman; 53.2% reported a moderately or very satisfactory current relationship, and 53.2% reported that the support they gave to the partner during the disease was moderately or very satisfactory.

Finally, with regard to psychological comorbidity, 61.2% of women had a possible case of anxiety, 25.2% had a possible case of depression, and 40.4% of women had decreased body image. In the male partners, it was found that 64.8% had a possible case of anxiety and 27.0% had a possible case of depression.

### 3.2. Quality of Life in Women and Their Partners

Women had an average score of 55.9 (*SD* = 8.6) in Physical Health and 48.2 (*SD* = 5.9) in Mental Health. Additionally, 76.6% had low Physical Health and 54.2% had low Mental health, according to the self-reported instrument SF-36. The male partners had an average score of 86.2 (*SD* = 5.0) in Physical Health and 79.2 (*SD* = 7.7) in Mental Health. All partners (100%) presented with acceptable Physical and Mental Health, according to the self-reported instrument SF-36.

### 3.3. Correlates of the Quality of Life of Women and Their Partners

As presented in Table 2, when analyzed simultaneously, the factors related to women’s Physical Health were months of surgery (non-standard coefficient = 2.24, *p* < 0.001, 95% CI 1.45, 3.75), no current treatments (non-standard coefficient = −6.94, *p* < 0.001, 95% CI −7.70, −2.02), completion of treatments (non-standard coefficient = 2.35, *p* < 0.001, 95% CI 0.61, 3.37), satisfaction with the current relationship with the partner (non-standard coefficient = 2.02, *p* < 0.001, 95% CI 0.58, 3.44), anxiety (non-standard coefficient = −3.87, *p* < 0.001, 95% CI −4.75, −0.73), depression (non-standard Coefficient = −3.83, *p* < 0.001, 95% CI −4.32, −0.84), and body image (non-standard coefficient = −0.93, *p* < 0.001, 95% CI −3.56, −0.30). For Mental Health, the following were significant: months of diagnosis (non-standard coefficient = 2.34, *p* < 0.001, 95% CI 1.06, 3.61), finished treatments (non-standard coefficient = 1.12, *p* < 0.001, 95% CI 0.34, 1.68), satisfaction with partner support during the disease (non-standard coefficient = 1.95, *p* < 0.001, 95% CI 0.34, 0.68), anxiety (non-standard coefficient = −2.67, *p* < 0.001, 95% CI −3.63, −3.27), depression (non-standard coefficient = −2.31, *p* < 0.001, 95% CI −3.14, −2.58), and body image (non-standard coefficient = 0.50, *p* < 0.001, 95% CI 0.37, 0.64).

From the set of potential predictors of the Physical Health of the partners, anxiety (non-standardized coefficient = −0.43, *p* < 0.001, 95% CI −3.60, −0.08) and depression (non-standardized coefficient = −0.15, *p* < 0.001, 95% CI −2.41, −0.29) were significant. Regarding Mental Health, anxiety (non-standard coefficient = −0.51, *p* <.001, 95% CI −1.03, −0.31) and depression (non-standardized coefficient = −0.41, *p* <.001, 95% CI −0.98, −0.03) were significant.

### 3.4. Sexual Satisfaction in Women and Their Partners

In women, there was an average total score of 7.5 (*SD* = 2.0) in sexual satisfaction and about 88.4% of women presented with sexual dysfunction, according to the self-reported instrument GRISS. In the male partners, an average total score of 6.92 (*SD* = 1.0) was found and 100% of the partners presented sexual dysfunction, according to the self-reported instrument GRISS.

### 3.5. Correlates of Sexual Satisfaction in Women and Their Partners

As shown in Table 3, when analyzed simultaneously, the factors related to women’s sexual satisfaction, were age (non-standard coefficient = −0.57, *p* < 0.001, 95% CI −1.06, −0.07), satisfaction with their relationship with their partner before the illness (non-standard coefficient = −0.03, *p* < 0.001, 95% CI −0.86, −0.35), anxiety (non-standard coefficient = 0.57, *p* < 0.001, 95% CI 0.45, 1.77), depression (non-standard coefficient = 0.15, *p* < 0.001, 95% CI 1.08, 4.08), and body image (non-standard coefficient = −0.42, *p* < 0.001, 95% CI −0.51, −0.33).

In the case of partners, from the set of potential predictors of the sexual satisfaction, the following were significant: psychological/psychiatric support (non-standard coefficient = −2.17, *p* < 0.001, 95% CI 1.02, 3.32), satisfaction with the current relationship with the partner (non-standard coefficient = −1.66, *p* < 0.001, 95% CI −2.03, −1.13), anxiety (non-standard coefficient = 1.00, *p* < 0.001, 95% CI 0.86, 1.13), and depression (non-standard coefficient = 1.23, *p* < 0.001, 95% CI 0.90, 1.10).

## 4. Discussion

The present study aimed to determine quality of life and sexual satisfaction, and its associated factors in women with breast cancer who underwent surgical treatments and their male partners. Of the female participants, 76.6% had low physical health and 54.2% had low mental health; of their male partners, none presented with poor physical and mental health. These data are congruent with studies that point to a compromise in the quality of life of women with breast cancer [9,35] but contrast with studies that indicate the presence of a lower quality of life of partners when compared with partners of healthy women [36].

When analyzing the factors associated with women’s physical health, it was found that time since surgery was a factor related to physical health: those who had longer time since surgery had better physical health, which is congruent with studies that show the tendency to adapt to the disease over time [35,37]. The time elapsed since diagnosis is also a protective factor of women’s mental health, which may be explained by the fact that the negative repercussions of treatment have a transitory trend and decrease over time [38]. Another predictor of physical health was not receiving current treatments. These results are in line with studies that have underlined the impact of chemotherapy, radiotherapy, and hormonal treatments on the quality of life of women with breast cancer due to its side effects such as nausea, vomiting, and alopecia [39,40]. The conclusion of the treatments was a predictor of physical health and the time elapsed since the conclusion of treatments was a predictor of mental health, which is consistent with the scientific literature and may be due to the disappearance of the side effects of the treatments [14]. Satisfaction with their current relationship with their partner was a predictor of women’s physical health, and satisfaction with the relationship during the disease was a predictor of mental health. These data are consistent with the literature that indicates that the quality of the affective relationship is crucial for the construction of a more positive quality of life of women with breast cancer [15]. Moreover, it was found that low levels of anxiety and depression were predictors of the physical and mental health of patients, which is in line with the literature, which suggests that quality of life is influenced by the presence of anxiety, depression, suicidal ideation, insomnia, and fear [16]. Finally, it was found that better body image resulted in a predictor of physical and mental health. The data found reinforce the results of a study that showed that changes in body image tend to be associated with the negative perspective of the disease, affecting quality of life [7].

As far as partners are concerned, only the lowest levels of anxiety and depression resulted in predictors of their physical and mental health. These data are aligned with studies that have shown a significant correlation between the psychological comorbidity of partners and their quality of life [36].

Regarding sexual satisfaction, it was found that 88.4% of women and 100% of partners presented sexual dysfunction. These results agree with the scientific literature, which is unanimous in the presentation of alterations in the sexual life of women with breast cancer, reaching an interruption in sexual life [41]. Some explanations for this are associated with the physical or psychological issues resulting from altered body image and pleasure sensations associated with breast stimulation, or side effects of surgical treatment, such as hair loss, weight gain, and scarring [11]. In addition, the present results suggest that partners also go through a process of adaptation to the new body image of their partners and restrict their sexual needs due to the psychological suffering of the partner and due to the fear of causing her some kind of physical suffering or reviving memories of the change in breast shape [42]. However, this finding must be interpreted with caution, due to research demonstrating that around 50% of men could have some degree of erectile dysfunction beforehand [43]. Future studies assessing the presence of a previous sexual dysfunction or physical condition are needed.

In the current study, an older age in the women was a predictor of greater sexual satisfaction. These results are consistent with research that shows that younger mastectomized women have a higher risk of developing sexual dysfunction and greater difficulty in adapting to their new body image [23]. Another predictor of women’s sexual satisfaction was greater satisfaction with their relationship with their partner before the disease because the understanding of the partner, the existence of a strong bond, and assertive communication help in the management of the process of sexual renegotiation that follows the oncological experience [10]. The predictors of sexual satisfaction were also a lower level of anxiety and depression, which is congruent with previous studies [21] due to the fact that anxiety interferes with the attention given to physiological responses and that depression decreases interest in sexuality. Finally, a better body image was a predictor of greater sexual satisfaction, which is congruent with the literature [22,23].

Among the predictors of sexual satisfaction of male partners, it was found that receiving psychological/psychiatric support was associated with sexual satisfaction. Psychological support is important for better acceptance of the woman’s body image and greater sexual satisfaction. In addition, partners who were more satisfied with their current relationship with their partner were more likely to experience sexual satisfaction. A strong bond between both and assertive communication helps the couple to determine the appropriate factors needed for sexual satisfaction [10,24,44]. Finally, lower levels of anxiety and depression were predictors of sexual satisfaction in partners. These data are aligned with studies that show the impact of anxiety and depression on sexual satisfaction [21].

### 4.1. Implications

There are important implications derived from this study for research and clinical practice, including the need to prioritize the identified factors in the treatment of women undergoing breast cancer treatment and their partners. Specifically, the results may help health providers understand the importance of discussing changes in body image and sexuality with their patients and their partners to ensure a high quality of life and sexual satisfaction.

### 4.2. Limitations 

A convenience sample (women of the Portuguese League Against Cancer) was used. However, the representativeness of the sample may be equivalent to a Portuguese national sample because this institution has huge recognition, the sample encompasses people from all regions of Portugal, and the vast majority of women who go through oncological processes are contacted by this institution. In addition, the results only reported data from self-reported instruments (no examination or interview was conducted, nor was any diagnosis made of any physical illness, mental disorder, or sexual dysfunction). This may have skewed the obtained results, which reflected perceived physical and mental health, and perceived sexual satisfaction. Although addressing sexual satisfaction through a questionnaire was the least invasive methodology, other methodologies for the assessment may be included in futures studies. Possible previous physical condition of the male partners was not specifically assessed. Although in this sample, the partners obtained a high average score for Physical Health Summary and 100% presented acceptable physical health in the SF-36 self-reported instrument, a specific evaluation may be considered in future studies. Finally, this study was conducted in Portugal, so the results found may not be generalizable to other countries.

## 5. Conclusions

The results of this study suggest that interventions aimed at increasing quality of life and sexual satisfaction in women with breast cancer who underwent surgical treatments and in their partners are needed. Emotional and sexual counseling during cancer treatment should include the partner.

## Figures and Tables

**Table 1:** Sociodemographic, clinical, relationship and therapeutic involvement, psychological comorbidity, and body image variables.

Socio-Demographic Variables (Women)	*n*	%
Age		
*M*	50.6	-
*SD*	12.6	-
Range	20–79	-
Education		
1st/2nd cycle	210	54.0
Up to secondary school	102	26.2
Higher education	77	19.8
Professional status		
Employed	240	61.7
Unemployed/retired	149	38.3
Civil status		
Single	10	2.6
Married	379	97.4
Have children		
Yes	323	83.0
No	66	17.0
Number of children		
*M*	1.0	-
*SD*	0.9	-
Range	0–4	-
Socio-demographic variables (men)	*n*	%
Age		
*M*	53.7	
*SD*	12.2	
Range	30–82	
Education		
1st/2nd cycle	226	61.7
Up to secondary school	61	16.7
Higher education	79	21.6
Professional status		
Employed	215	58.7
Unemployed/retired	151	41.3
Diagnostic and treatment variables (women)	*n*	%
Time of diagnosis (months)		
*M*	53.6	-
*SD*	24.2	-
Range	12–96	-
Time of surgery (months)		
*M*	47.4	-
*SD*	22.0	-
Range	11–96	-
Type of surgery		
Conservative surgery	124	31.9
Mastectomy and breast reconstruction	265	68.1
Time of breast reconstruction (months)		
*M*	18.0	-
*SD*	24.7	-
Range	0–96	-
Type of initial treatment		
Initial chemotherapy	85	21.9
Initial radiotherapy	63	16.1
Combination of several treatments	241	62.0
Conclusion of treatments		
Yes	280	72.0
No	109	28.0
Time to treatment completion (months)		
*M*	28.2	-
*SD*	23.7	-
Range	0–84	-
Current treatment type		
Current chemotherapy	49	12.6
Current radiotherapy	3	0.8
Current hormone therapy	39	10.0
Combination of various current treatments	18	4.6
Side effects		
Alopecia	275	71.2
Weight loss	35	8.7
Weight gain	15	3.8
Change in skin texture	56	14.3
Swelling of the arm	8	2.0
Menopause		
Yes	228	58.6
No	161	41.4
Psychological/psychiatric follow-up		
Yes	284	73.0
No	105	27.0
Treatment variables (men)	*n*	*%*
Psychological/psychiatric follow-up		
Yes	68	18.6
No	298	81.4
Clinical data of the relationship and engagement (women)	*n*	%
Relationship before the illness		
Not at all/not very satisfactory	104	26.2
Moderately/very satisfactory	285	73.8
Current relationship		
Not at all/not very satisfactory	141	36.2
Moderately/very satisfactory	248	63.8
Support from partner		
Not at all/not very satisfactory	165	42.4
Moderately/very satisfactory	224	57.6
Sexuality approached by health professionals		
Yes	61	15.7
No	328	84.3
Involvement in choice of treatment		
Yes	100	25.7
No	289	74.3
Possibility of sharing experience with other women		
Yes	75	19.3
No	314	80.7
Clinical data of the relationship and engagement (men)	*n*	%
Relationship before the illness		
Not at all/not very satisfactory	158	46.5
Moderately/very satisfactory	208	53.5
Current relationship		
Not at all/not very satisfactory	159	46.8
Moderately/very satisfactory	207	53.2
Support given to partner		
Not at all/not very satisfactory	159	46.8
Moderately/very satisfactory	207	53.2
Psychological comorbidity variables (women)	*n*	%
Anxiety		
*M*	9.2	
*SD*	4.7	
Range	0–21	
No case	151	38.8
Case	238	61.2
Depression		
*M*	6.8	
*SD*	5.2	
Range	0–21	
No case	291	74.8
Case	98	25.2
Body image variables (females)		
*M*	53.6	
*SD*	24.2	
Range	12–96	
Worst relationship with the body	157	40.4
Better relationship with the body	232	59.6
Psychological comorbidity variables (men)	*n*	%
Anxiety		
*M*	8.7	
*SD*	3.6	
Range	0–18	
No case	129	35.2
Case	237	64.8
Depression		
*M*	5.3	
*SD*	3.8	
Range	0–18	
No case	267	73.0
Case	99	27.0

**Table 2:** Predictors of quality of life.

Quality of Life Predictors	Non-Standardized Coefficients		Standard Coefficients	*t*	*p*	95% Confidence Interval for B	
	B	Typical Error	Beta			Lower Limit	Upper Limit
Predictors of women’s Physical Health							
Months since surgery	2.24	0.71	0.19	3.15	<0.001	1.45	3.75
Current treatments No treatments Treatments	−6.94	3.33	−0.14	3.14	<0.001	−7.70	−2.02
Conclusion of treatments	2.35	0.74	0.13	−4.08	<0.001	0.61	3.37
Satisfaction with current relationship with partner Very satisfied Not at all satisfied	2.02	0.28	0.42	6.99	<0.001	0.58	3.44
Anxiety	−3.87	0.32	−0.74	6.32	<0.001	−4.75	−0.73
Depression	−3.83	0.47	−0.69	6.75	<0.001	−4.32	−0.84
Body image	−0.93	0.08	−0.63	6.35	<0.001	−3.56	−0.30
Predictors of women’s Mental Health							
Months since diagnosis	2.34	0.64	0.27	3.653	<0.001	1.06	3.61
Treatment completion (months)	1.12	0.08	0.22	6.03	<0.001	0.34	1.68
Satisfaction with the partner’s support during the illness Very satisfied Not at all satisfied	1.95	0.29	0.50	7.49	<0.001	0.34	0.68
Anxiety	−2.67	0.09	−0.65	−8.69	<0.001	−3.63	−3.27
Depression	−2.31	0.12	−0.44	−7.71	<0.001	−3.14	−2.58
Body image	0.50	0.15	0.32	7.48	<0.001	0.37	0.64
Predictors of partners’ physical health							
Anxiety	−0.43	0.54	−0.35	−3.50	<0.001	−3.60	−0.08
Depression	−0.15	0.32	−0.42	−2.89	<0.001	−2.41	−0.29
Predictors of partners’ mental health							
Anxiety	−0.51	0.47	−0.49	−4.20	<0.001	−1.03	−0.31
Depression	−0.41	0.12	−0.52	−3.54	<0.001	−0.98	−0.03

**Table 3:** Predictors of sexual satisfaction.

Predictors of Sexual Satisfaction	Non-Standardized Coefficients		Standard Coefficients	*t*	*p*	95% Confidence Interval for B	
	B	Typical Error	Beta			Lower Limit	Upper Limit
Predictors of women’s sexual satisfaction							
Age	−0.57	0.25	−0.08	−2.26	<0.001	−1.06	−0.07
Satisfaction with relationship with partner before illness Very satisfied Not at all satisfied	−0.03	−0.60	−0.12	−4.74	<0.001	−0.86	−0.35
Anxiety	0.57	1.11	0.23	0.24	<0.001	0.45	1.77
Depression	0.15	2.58	0.75	0.13	<0.001	1.08	4.08
Body image	−0.42	0.04	−0.60	−9.27	<0.001	−0.51	−0.33
Predictors of men’s sexual satisfaction							
Psychological/psychiatric support	−2.17	0.58	0.13	3.71	<0.001	1.02	3.32
Satisfaction with current relationship with partner Very satisfied Not at all satisfied	−1.66	0.18	−0.33	−8.94	<0.001	−2.03	−1.30
Anxiety	1.00	0.06	0.55	14.77	<0.001	0.86	1.13
Depression	1.23	0.11	0.47	12.25	<0.001	0.90	1.10

## Data Availability

The data presented in this study are available from the corresponding author upon request. The data are not publicly available due to confidentiality issues.
